# P2RX7 in Dopaminergic Neurons of Ventral Periaqueductal Gray Mediates HTWP Acupuncture-Induced Consciousness in Traumatic Brain Injury

**DOI:** 10.3389/fncel.2020.598198

**Published:** 2021-01-13

**Authors:** Huiling Tang, Siru Qin, Wei Li, Xuyi Chen, Luis Ulloa, Qiumei Zhu, Baohu Liu, Yinan Gong, Yadan Zhao, Songtao Wang, Shanshan Li, Yongming Guo, Zhifang Xu, Yi Guo

**Affiliations:** ^1^Acupuncture Research Center, Tianjin University of Traditional Chinese Medicine, Tianjin, China; ^2^Department of Neurosurgery, Characteristic Medical Center of Chinese People's Armed Police Force, Tianjin, China; ^3^Institution of Brain Trauma and Neurology Disease of People's Armed Police Forces, Tianjin, China; ^4^Tianjin Key Laboratory of Neurotrauma Repair, Tianjin, China; ^5^Department of Anesthesiology, Center of Perioperative Organ Protection, Duke University Medical Center, Durham, NC, United States; ^6^Luoding Hospital of Traditional Chinese Medicine, Guangdong, China; ^7^Department of Rehabilitation, Wangjing Hospital, China Academy of Chinese Medical Sciences, Beijing, China; ^8^School of Acupuncture & Moxibustion and Tuina, Tianjin University of Traditional Chinese Medicine, Tianjin, China; ^9^School of Traditional Chinese Medicine, Tianjin University of Traditional Chinese Medicine, Tianjin, China

**Keywords:** acupuncture, traumatic brain injury, neural projection, dopamine, P2RX7, ventral periaqueductal gray, hand 12 *Jing*-well points

## Abstract

The induction of a coma by traumatic brain injury (TBI) is a crucial factor for poor clinical prognoses. We report that acupuncture at the hand 12 *Jing*-Well points (HTWP) improved consciousness and neurologic function in TBI rats. Gene chip analyses showed that HTWP acupuncture mostly activated genes modulating neuronal projections (*P2rx7, P2rx3, Trpv1, Tacr1*, and *Cacna1d*), protein secretion (*Exoc1, Exoc3l1, Fgb*, and *Fgr*), and dopamine (DA) receptor D3 (*Drd3*) in the ventral periaqueductal gray (vPAG), among which the expression rate of *P2rx7* was the most obviously increased. Acupuncture also increased the expression and excitability of DA and P2RX7 neurons, and the DA neurons expressed P2RX7, P2RX3, and TRPV1 in the vPAG. Intracerebroventricular administration of P2RX7, P2RX3, or TRPV1 antagonists blocked acupuncture-induced consciousness, and the subsequent injection of a P2RX7 antagonist into the vPAG nucleus also inhibited this effect. Our findings provide evidence that acupuncture alleviates TBI-induced comas via DA neurons expressing P2RX7 in the vPAG, so as to reveal the cellular and molecular mechanisms of the improvement of TBI clinical outcomes by HTWP acupuncture.

## Highlights

- Acupuncture at the hand 12 *Jing-W*ell points (HTWP) promotes awakening in TBI rats.
- HTWP acupuncture activates a specific neural network in the brainstem and ascending reticular activating system of TBI rats.
- HTWP acupuncture activates P2RX7 dopaminergic neurons in the ventral periaqueductal gray.

## Introduction

Traumatic brain injury (TBI) is a major challenge for modern medicine; it affects about 55 million people worldwide, with over 27 million new cases reported in 2016 (GBD 2016 Traumatic Brain Injury Spinal Cord Injury Collaborators, 2019). Despite multiple efforts to develop effective treatments, there are still no first-line responder therapeutic strategies to ameliorate TBI pathogenesis and progression. TBI-induced comas are a key factor in poor clinical prognoses, including death (Hutchinson et al., 2016). The pathophysiological mechanisms that induce a coma include injury of the neuronal circuits of the ascending reticular activating system (ARAS), especially in the pons and mesencephalon (Jang et al., 2019). Thus, neurostimulators are often surgically implanted into the brainstem or spinal cord to induce ARAS stimulation and thereby promote consciousness in comatose patients. Although some pilot clinical trials have shown that these surgical procedures are somewhat efficacious, they can only be performed when the patient's vital signs are stable. Therefore, there remains a need for safe and practical therapeutic strategies that first responders can apply urgently at the hyperacute stage.

Acupuncture is an ancient traditional Chinese medicine (TCM) therapy that is presently used worldwide to treat millions of people with multiple disorders. Currently, acupuncture is endorsed by the World Health Organization for improving postoperative recovery, osteoarthritis, migraine, joint pain, stroke, and posttraumatic stress (Wang et al., 2018). Recent clinical trial data and meta-analyses showed that acupuncture improves cognition and other clinical outcomes of central nervous system injuries, including TBI (Zhu et al., 2019), stroke (Hung et al., 2019), and spinal cord injury (Xiong et al., 2019). In emergency TCM, the use of acupuncture at the hand 12 *Jing-*Well points (HTWP) was first documented in the Inner Canon of Huangdi ancient Chinese medicine treatise to promote consciousness in acute comatose patients. HTWP acupuncture involves inserting a needle perpendicular to the skin at the 12 acupoints located at the tips of the fingers: *Shaoshang* (LU11), *Shangyang* (LI1), *Zhongchong* (PC9), *Guanchong* (TE1), *Shaochong* (HT9), and *Shaoze* (SI1). We previously reported that HTWP acupuncture promotes consciousness in comatose patients and improves the neurological score of patients suffering from TBI, stroke, or carbon monoxide poisoning (Zhu et al., 2019; Yu et al., 2020). Multiple experimental studies also showed that HTWP acupuncture alleviates brain edema and improves tissue perfusion, blood–brain barrier integrity, and nerve function in rodents with TBI and strokes (Yu et al., 2017; Li et al., 2019, 2020). Despite these promising results, the mechanisms by which HTWP acupuncture induces consciousness in acute coma patients have not been elucidated. In this study, we investigated how HTWP acupuncture induces consciousness by decreasing the comatose period in TBI rats. HTWP acupuncture was performed on experimental rodents after severe TBI to determine its effects on key signaling pathways in the neuronal networks of the brainstem and nuclei of the ARAS. These studies can provide critical insights into novel clinic applications of HTWP acupuncture to promote consciousness in patients with pathologies affecting the central nervous system, such as TBI.

## Methods

### Animals

We carried out all experimental procedures in strict accordance with the Tianjin University of Traditional Chinese Medicine guidelines for the care and use of laboratory animals. The study protocol was approved by the Animal Care and Use Committee of Tianjin University of Traditional Chinese Medicine (Permit Number: TCM-LAEC2019058). All surgeries were performed on animals under 10% chloral hydrate anesthesia, and thorough measures were taken to minimize suffering. Any rats that died during surgery were excluded from the study.

Adult male Sprague-Dawley rats weighing 280–320 g were purchased from the Experimental Animal Center of Beijing Wei Tong Li Hua Experimental Animal Technology Co., Ltd. (Beijing, China). The license number was SCXK (Beijing) 2016-0006. All animals were maintained in cages under a 12/12-h light/dark cycle at 24–26°C and a controlled relative humidity of 50–60%, and they were allowed free access to food and water.

### Establishment of a Severe TBI Animal Model

**Figure 1:**
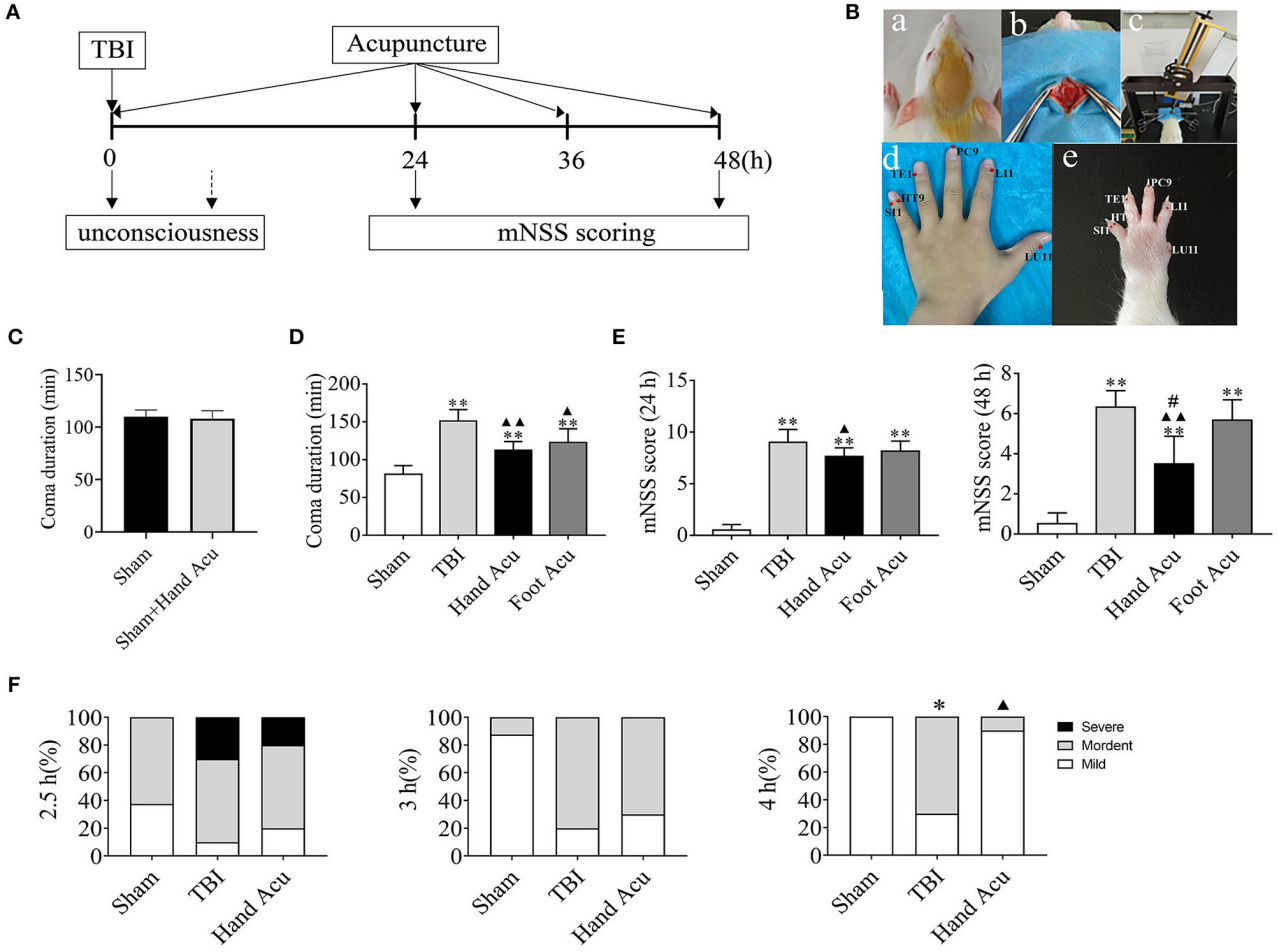
HTWP acupuncture improves consciousness in TBI rats. **(A)** Diagram of acupuncture treatments and modified neurological severity score (mNSS). HTWP acupuncture was performed after TBI (0 h) and repeated at 24 and 48 h. mNSS scoring was assessed after the acupuncture treatments at 24 and 48 h. **(B)** Experimental design: anesthesia (a), skull exposure (b), electronic controlled cortical impactor (eCCI) and TBI impact (c), and hand 12 *Jing-*Well points (HTWP) (d–e). **(C)** The effect of acupuncture at HTWP on choral hydrate induced unconsciousness (*n* = 8/group; student's *t*-test). **(D)** TBI-induced coma duration after acupuncture (*n* = 6/group; one-way ANOVA). **(E)** mNSS at 24 or 48 h post-TBI in hand vs. control foot acupuncture, or (*n* = 6/group; one-way ANOVA). **(F)** I–VI consciousness scale assessment (mild, moderate, or severe) at the indicated post-TBI time points (*n* = 10/group; Kruskal–Wallis H) in sham and TBI rats treated without or with HTWP (hand) or FTWP (foot) acupuncture. Error bars represent mean + SEM; ^*^P < 0.05, ^**^P < 0.01 vs. Sham; ^▴^*P* < 0.05, ^▴▴^*P* < 0.01 vs. TBI; ^#^*P* < 0.05 vs. Foot Acu.

As shown in Figure 1B, rats were anesthetized intraperitoneally with 10% chloral hydrate (0.35 ml/100 g) then placed on a stereotactic frame and fixed in the prone position. A midline longitudinal incision was made on the head to expose the skull. A right parietal craniotomy (5 mm in diameter, 3.5 mm from the sagittal suture, and 2.5 mm to the right of the midline) was carried out with a craniocerebral operational drill, while keeping the cerebral dura intact. We employed a cortical contusion injury model of TBI in the rat (Custom & Design Company, Richmond, VA, USA) (Romine et al., 2014). Injury was induced via an electronic controlled cortical impactor (eCCI) with a 3-mm-diameter tip operated at a rate of 4 m/s, depth of 3 mm, and dwell time of 120 ms; then the scalp was sutured. Sham-operated animals were anesthetized as above, and the right parietal craniotomies were surgically prepared without subsequent cortical contusion (Tu et al., 2016).

### HTWP Acupuncture Treatment

Standard comparative anatomy was used to locate the acupoints in the rats according to records of human anatomical acupoints (Figure 1B d,e). HTWP acupuncture was performed by inserting a needle perpendicular to the skin at the 12 HTWP at the tips of the fingers, named LU11, LI1, PC9, TE1, HT9, and SI1 acupoints and retaining for 5 min. The specificity of this treatment was confirmed by performing alternative acupuncture at the foot 12 *Jing*-Well points (FTWP), named *Yinbai* (SP1), *Dadun* (LR1), *Lidui* (ST45), *Zuqiaoyin* (GB44), *Zhiyin* (BL67), and *Yongquan* (KI1). Our procedures followed the national GB/T 21709.4-2008 acupuncture technical operation standard, part 4. To ensure that the process ran smoothly, the rats were restrained using a soft cloth, and disposable acupuncture needles (diameter of 0.35 mm and length of 25 mm, Hanyi TCM, Beijing, China) were inserted perpendicular to the skin surface to a 1-mm depth bilaterally around the HTWP. HTWP acupuncture was performed immediately after the TBI procedure and repeated 24 and 48 h later.

### Determination of Coma Duration

The coma duration was defined as the period of time from when the anesthesia took effect to the recovery of consciousness. After establishing the model, we observed the coma duration of the rats in real time and recorded the timepoints at which the rats regained consciousness. The conscious recovery level of the rats was determined through the appearance of the corneal reflex, righting reflex, and toe response. Specifically, the conscious recovery of an autonomous posture from the supine position and self-conscious crawling were the criteria used to determine the consciousness of the rats.

### Evaluation of Neurological Deficits

The modified neurological severity score (mNSS) was determined at 24 and 48 h post-TBI by blinded and trained observers. The assessment content included the alteration of motor and sensory functions, reflexes, and behaviors, with the highest score of 18 indicating severe neurological deficit (Chen et al., 1996, 2001a,b).

### I–VI Consciousness Scale

At 2.5, 3, and 4 h after the injury, the degree of consciousness was evaluated on a scale of I–IV based on sensory and motor functions (I–VI consciousness scale) (Stephens and Levy, 1994). The levels of consciousness were scored as follows: Level I, normal activity, as seen before surgery; Level II, decreased activity; Level III, decreased activity accompanied by motor incoordination; Level IV, righting reflex was elicited, and the animal could stand up; Level V, the righting reflex was absent, but the animal reacted to pain; and Level VI, the animal showed no reaction to pain. Rats with consciousness levels of V or VI for at least 30 min were deemed to be in a comatose state and were used in the intervention study. Each level was assigned a score between 1 and 6, with 1–2 representing a mild disturbance of consciousness, 3–4 representing a moderate disturbance of consciousness, and 5–6 representing a severe disturbance of consciousness. The constituent ratio (%) of each timepoint among the three groups was calculated to evaluate the effectiveness of acupuncture in promoting awakening and improving sensory and motor function.

### Gene Chip Analysis

At 1 h after modeling (*n* = 5), the rats were sacrificed by dislocation, and the brainstem and TBI-damaged cortex tissues were isolated and immediately frozen in liquid nitrogen (−195.79°C), then transferred to a −80°C freezer until they were used in further experiments.

The 1-h brainstem samples (*n* = 5) were extracted and purified, and the qualities of the total RNA were tested using a standard Affymetrix protocol according to Shanghai Biotechnology Co. (Shanghai, China). The raw chip data are accessible from the BioProject ID PRJNA 663342 in the public database of the NCBI BioProject^1^. Briefly, total RNA was isolated, and the RNA integrity number (RIN) was checked for RNA integration (Wang et al., 2013). Only RNA with an RIN value > 7.0 and a 28S/18S ratio > 0.7 was used for microarray analyses. The Affymetrix rat Genome 230 2.0 array was used to perform whole-genome microarray analysis using a GeneChip Scanner 3000 (Cat^#^ 00-00212, Affymetrix, Santa Clara, CA, US) and analyzed using Command Console Software 4.0 (Affymetrix) and MAS 5.0 algorithm (Gene Spring Software 11.0; Agilent Technologies, Santa Clara, CA, US) (Wang et al., 2013; Li et al., 2014). Then, the differential genes were screened using a threshold method, and the genes with a linear fold change ≥2 or ≤ 0.5 were considered differentially expressed genes. Additionally, the data was further analyzed according to the respective manufacturer's protocol (Wang et al., 2013). The selection criteria were the number of different genes matching a certain term in GO ≥2 and a *P* ≤ 0.05. The GO terms obtained were sorted in descending order of size according to the value of the enriching factor and considering the top 30 pathways.

### Reverse Transcription-Quantitative Polymerase Chain Reaction

The 1-h brainstem sample tissue total RNA was extracted as mentioned in section **2.7**, while the 1-h TBI-damaged cortex tissue samples (*n* = 5) were frozen in liquid nitrogen to be ground in a cold mortar. The total RNA was extracted using TRIzol reagent (Thermo Fisher Scientific China, Shanghai, China) in accordance with the manufacturer's protocol (Liu et al., 2016). The RNA concentration was measured with an Agilent Bioanalyzer 2100 (Agilent Technologies), and total RNA was used for reverse transcription with the PrimeScript RT reagent kit (Takara Bio, Inc., Otsu, Japan) following the manufacturer's protocol. Briefly, 1 μg of RNA template was used to synthesize the first-strand cDNA for each reaction mixture, and RT was conducted at 37°C for 15 min, followed by 5 s at 85°C to terminate the reaction.

The cDNA was amplified using SYBR Premix ExTaq II (Takara Bio, Inc.) and a reverse transcription-quantitative polymerase chain reaction (RT-qPCR) procedure, according to the manufacturer's protocol. The ABI QuantStudio 3 Time PCR System (Applied Biosystems; Thermo Fisher Scientific, Inc., US) was used to perform RT-qPCR under the following conditions: 95°C for 30 s, followed by 40 cycles of 95°C for 5 s and 60°C for 30 s, and finally a melting curve stage (95°C for 15 s, 60°C for 1 min, and 95°C for 15 s). The associated primers were synthesized by Shanghai Shenggong Biological Engineering Co., Ltd. and are listed in Supplementary Table 1. Relative gene expression was calculated using the double-standard curve method. Three independent experiments were repeated.

### Immunofluorescence Staining and Image Analysis

The rats were perfused transcardially with 4% paraformaldehyde 1 h after TBI, and tissue samples were immersed in 4% paraformaldehyde for 24 h. After 72 h of replacement with 20% sucrose solution, the OCT-embedded brain samples were sliced to a thickness of 10 μm with a Leica frozen slicer (1850). Briefly, the brain slices were rinsed with PBS and high-temperature antigen-repaired. The slices were incubated in 5% fetal bovine serum for 30 min to block the non-specific binding of immunoglobulins. Then, the sections were incubated with the primary antibodies rabbit anti-P2RX7 (1:2000, Abcam, MA, USA), rabbit anti-P2RX3 (1:500, Abcam), rabbit anti-TRPV1 (1:500, Abcam), and mouse anti-dopamine (1:200, Abcam) overnight at 4°C. After a PBS rinse, the sections were incubated in the dark with fluorescently labeled secondary antibodies at an appropriate dilution at 37°C for 1 h, followed by a PBS wash. After being counterstained with DAPI, incubated for 10 min, and covered with an anti-fade mounting medium, the sections were observed under a Leica (DMi8) inverted fluorescence microscope and images were taken.

For the image analysis, 8-bit images were captured with the Tissue FAXS slide scanning system and analyzed using Tissue Quest software (Tissue Gnostics, Vienna, Austria). We quantified the total number of cells based on the number of DAPI-positive nuclei and the number of cells showing green fluorescent protein Alexa Fluor 488 (DA) and red fluorescent protein Alexa Fluor 594 (P2RX7, P2RX3, and TRPV1) fluorescence above a particular background threshold. In brief, DAPI-positive nuclei were segmented using an algorithm that quantifies the intensity of pixels over the number of nuclei and creates a mask distinguishing each nucleus. Then, the Alexa Fluor 488 and Alexa Fluor 594 signals were plotted against the nuclear DAPI signal and against each other to create two-dimensional scattergrams. We then quantified only those positive signals associated with cells defined by DAPI-positive nuclei. Populations of Alexa Fluor 488- and Alexa Fluor 594-positive cells were further characterized by first thresholding for known levels of positive fluorescence based on controls and gating for single- or double-stained populations representing the cells of interest.

### Lateral Intracerebroventricular Injection of Antagonist

The rats were randomly assigned into the following seven groups (*n* = 6 rats per group): TBI + i.c.v DMSO, TBI + i.c.v DMSO + Acu, TBI + P2RX7 antagonist (A-438079 hydrochloride hydrate, A9736) + Acu, TBI + TRPV1 antagonist (SB366791, S0441) + Acu, TBI + i.c.v saline, TBI + i.c.v saline + Acu, and TBI + P2RX3 antagonist (sodium A-317491 salt hydrate, A2979) + Acu. The modeling method was the same as before. The rats' heads were secured using stereotaxic instruments, with the head held in a flat skull position. After leveling the rat brain, the coordinates of bregma were determined using a rat brain atlas. The microinjection coordinates were anteroposterior −0.9 mm, mediolateral + 1.5 mm, and dorsoventral −4.2 mm (**Figure 6A**). A 10-μL Hamilton microliter syringe was used to inject 5 μL of test solution. The concentrations of the antagonists P2RX7, P2RX3, and TRPV1 were 8.765 nmol/5 μL (Chu et al., 2012), 25 nmol/5 μL (Liu et al., 2017), and 1 nmol/5 μL (Palhares et al., 2017), respectively. A simple mark was made at the point where the syringe touched the surface of the skull. A hole of 1 mm diameter was carefully drilled with an electric cranial drill at the mark. The microinjector was filled 5 μL antagonist, fixed on the brain stereotactic apparatus, and then moved to the target bone hole. The syringe was slowly lowered to the target site for 5 min and was retained at the target place for an additional 5 min before beginning the injection (0.5 μL per minute), then it was slowly removed over a 5-min period. TBI impact was performed 30 min after injection, immediately followed by acupuncture at the HTWP. The coma duration was observed in real time, and we statistically analyzed differences compared with the Hand Acu group to determine the impact of P2RX7/P2RX3/TRPV1 on the HTWP acupuncture awakening effect. All the experimental rats were sacrificed by dislocation 6 h after modeling (**Figure 6B**).

### Ventral Periaqueductal Gray Intranuclear Injection of Antagonist

Rats were randomly assigned to the following four groups (*n* = 6 per group): TBI + DMSO, TBI + P2RX7 antagonist (A-438079 hydrochloride hydrate, A9736), TBI + DMSO + Acu, TBI + P2RX7 antagonist + Acu. After leveling the rat brain, the microinjection coordinates of bregma determined using a rat brain atlas were anteroposterior −7.8 mm, mediolateral ± 0.35 mm, dorsoventral −6.8 mm. P2RX7 was used at 1 nmol/μL (Hu et al., 2020). A 1-μL Hamilton microliter syringe was filled with 0.25 μL antagonist, which was injected at a rate of 0.05 μL/min into the ventral periaqueductal gray (vPAG) nucleus. The injection, modeling, and intervention methods were as before. Then, the coma durations were real-time observed and analyzed to determine the effect of injecting the P2RX7 antagonist into the vPAG nucleus on coma duration. All the experimental rats were sacrificed by dislocation 6 h after modeling.

### Statistical Analysis

The quantitative data are presented as the mean ± standard error of the mean (SEM) of at least three experimental repeats. Data are presented as mean ± SEM. SPSS 21.0 statistical software was used for all statistical analyses, and GraphPad Prism (GraphPad, San Diego, CA, USA) was used for mapping. The significance of the differences between groups was evaluated using student's *t*-test for two groups and one-way analysis of variance (ANOVA) for multiple-group comparison. Kruskal–Wallis H was used for the distribution of ranked data. *P* < 0.05 was considered to indicate a statistically significant difference.

## Results

### HTWP Acupuncture Improved Consciousness and Neurologic Function in TBI Rats

First, we analyzed whether HTWP and FTWP acupuncture affects coma duration and improves the mNSS of TBI rats following the experimental timeline (Figure 1A). Rodents underwent surgical sham or TBI induced with an eCCI followed by HTWP acupuncture (hand Acu) or control FTWP acupuncture (foot Acu). HTWP acupuncture was performed by stimulating the 12 HTWP located at the tips of the fingers, including LU11, LI1, PC9, TE1, HT9, and SI1 acupoints. The specificity of this treatment was determined by performing an alternative control acupuncture treatment at the 12 FTWP, including SP1, LR1, ST45, GB44, BL67, and KI1 (Figure 1B). On the basis of confirming that acupuncture had no promoting effect on coma caused by chloral hydrate (Figure 1C), we observed whether there were any differences between HTWP and FTWP acupuncture in promoting the waking effect (Figure 1D). The results showed that the average unconsciousness period lasted 80.67 ± 4.63 min in sham animals as a result of the anesthesia. TBI dramatically doubled this coma duration to an average of 151.17 ± 6.24 min. HTWP acupuncture was effective at reducing the coma duration to 112.50 ± 4.67 min (*P* < 0.01), and FTPW acupuncture reduced it to an average of 122.67 ± 7.45 min (*P* < 0.05) compared with TBI, but there was no statistical difference between the two groups. TBI increased the coma duration by around 70 min compared with the sham group, whereas HTWP acupuncture reduced this effect by 55% to 32 min (Figure 1D). After the animals recovered consciousness, we further analyzed the neurological damage by measuring the mNSS score after acupuncture treatment at 24 and 48 h (Figure 1E). HTWP acupuncture significantly improved the neurological scores at 24 h, and the effect was even more statistically significant at 48 h post-TBI. By contrast, FTWP acupuncture failed to improve the neurological score. Together, these results indicate that HTWP acupuncture significantly improved consciousness and neurologic function in TBI rats through a specific mechanism compared with FTWP acupuncture.

We additionally applied the I–VI consciousness scale after the rats had revived to further assess the effect of HTWP acupuncture on consciousness at 2.5, 3, and 4 h post-TBI (Figure 1F). We observed that, although HTWP acupuncture slightly improved consciousness at all observation points in this experiment, the effects became statistically significant (*P* < 0.05 vs. TBI; *n* = 5/group) at 4 h post-TBI, with HTWP acupuncture decreasing moderate damage to 10% compared with a reduction to 70% in the TBI group without acupuncture.

### HTWP Acupuncture Activated the Pathways of Neural Projection and Protein Secretion in the Brainstem of TBI Comatose Rats

**Figure 2:**
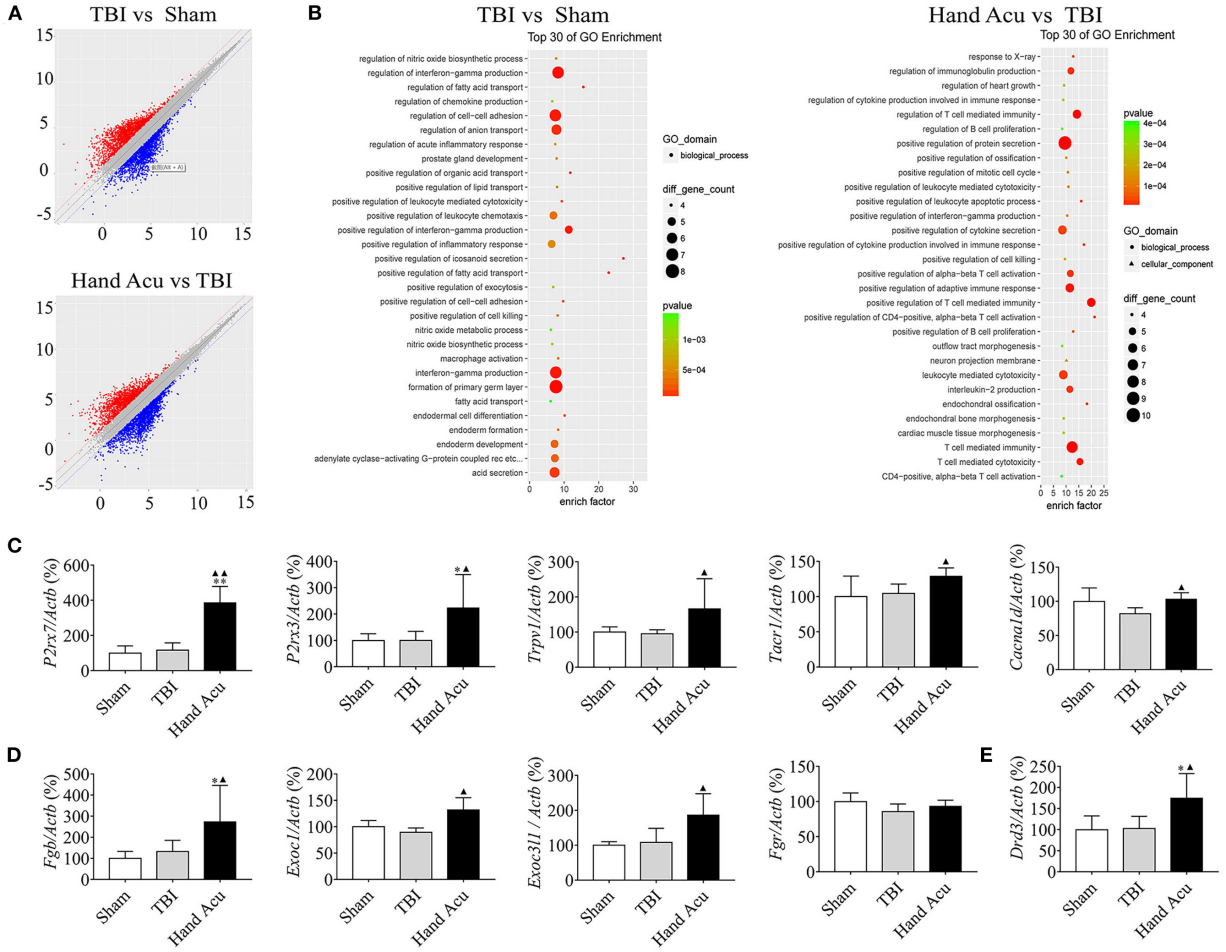
HTWP acupuncture activates the pathways of neural projection and protein secretion in the brainstem. **(A)** Differential gene scatter plots (red and blue plots represent up- and downregulated genes, respectively). **(B)** Top 30 of GO enrichment analysis. The larger dots represent higher number of genes. The following genes were analyzed (*n* = 5/group; one-way ANOVA). **(C–E)** Quantitative analysis of the gene expression of factors related to **(C)** neural projections: *P2rx7, P2rx3, Trpv1, Tacr1*, and *Cacna1d* or **(D)** protein secretion: *Exoc1, Exoc3l1, Fgb, Fgr*, and **(E)** *Drd3*. Error bars represent mean + SEM; *P < 0.05, **P < 0.01 vs. Sham; ^▴^*P* < 0.05, ^▴▴^*P* < 0.01 vs. TBI.

Multiple experimental and clinical studies have proven that TBI-induced comas are related to neuronal circuits of the ARAS, particularly those networks containing the multiple arousal-related nuclei of the brainstem, such as the vPAG (Jang et al., 2019). To determine the molecular mechanisms by which HTWP acupuncture induces consciousness, we analyzed differential gene expression in the brain stem. TBI modified the expression of 454 genes, increasing 193 and decreasing 261 gene expression levels, at 1 h post-trauma compared with the levels in sham animals. HTWP acupuncture modified the expression of 420 genes by increasing 149 and decreasing 271 gene expression levels at 1 h post-trauma compared with the TBI only group (Figure 2A). Among these, we identified 65 genes that were differentially expressed among the sham, TBI, and acupuncture groups (Supplementary Table 2). Differential gene GO pathway enrichment analysis revealed the major pathways modulated by HTWP acupuncture as compared with TBI-only animals (Table 1). These results indicate that TBI mainly activated inflammatory responses and cell adhesion molecules, while acupuncture modulated some of these inflammatory responses, the main effects of which were found at the cellular pathways regulating neural projection and protein secretion (Figure 2B).

**Table 1:** Top 30 items of GO enrichment from comparison of differential genes in acupuncture and TBI rats.

**Pathway**	***q* value**	**Gene count**	**Enrich factor**
Positive regulation of CD4-positive, alpha-beta T cell activation	0.001111	4	21.15
Positive regulation of T cell mediated immunity	0.00007437	6	19.52
Endochondral ossification	0.001957	4	18.13
Positive regulation of cytokine production involved in immune response	0.002134	4	16.92
Positive regulation of leukocyte apoptotic process	0.002298	4	15.86
T cell mediated cytotoxicity	0.001003	5	15.11
Regulation of T cell mediated immunity	0.0003899	6	13.84
Response to X-ray	0.003433	4	12.69
Positive regulation of B cell proliferation	0.003433	4	12.69
T cell mediated immunity	0.00009964	8	11.8
Regulation of immunoglobulin production	0.00208	5	11.54
Positive regulation of alpha-beta T cell activation	0.002215	5	11.33
Interleukin-2 production	0.002094	5	11.13
Positive regulation of adaptive immune response	0.001019	6	11.03
Positive regulation of leukocyte mediated cytotoxicity	0.004794	4	10.8
Positive regulation of mitotic cell cycle	0.004958	4	10.57
Positive regulation of interferon-gamma production	0.005252	4	10.36
Neuron projection membrane	0.005617	4	10.15
Positive regulation of ossification	0.005813	4	9.95
Positive regulation of cell killing	0.006537	4	9.4
Regulation of heart growth	0.006928	4	9.06
Endochondral bone morphogenesis	0.007197	4	8.91
Cardiac muscle tissue morphogenesis	0.007197	4	8.91
Positive regulation of protein secretion	0.00008556	10	8.87
Regulation of cytokine production involved in immune response	0.007532	4	8.75
Leukocyte mediated cytotoxicity	0.002263	6	8.46
Regulation of B cell proliferation	0.0088	4	8.32
Outflow tract morphogenesis	0.0088	4	8.32
Positive regulation of cytokine secretion	0.002614	6	8.1
CD4-positive, alpha-beta T cell activation	0.007303	4	8.06

We confirmed the most significant results by RT-qPCR gene quantification. Among the analyzed genes related to neural projection, acupuncture increased the expression of *P2rx7* by 231.40%, *P2rx3* by 123.08%, *Trpv1* by 74.97%, and *Tacr1* by 23.27% compared with in TBI animals. However, acupuncture increased the levels of *Cacna1d* by 26.38% to compensate for the inhibition induced by TBI (Figure 2C). Among the genes related to protein secretion, HTWP acupuncture was most effective at increasing the expression of *Fgb* (105.90%), *Exoc1* (47.31%), and *Exoc3l1* (72.48%) (Figure 2D). Our results also reveal that HTWP acupuncture induced a remarkable increase in *Drd3* expression (70.15%) in the vPAG (Figure 2E), which suggests that dopamine and dopaminergic neurons play critical roles in the mechanisms behind HTWP acupuncture treatment of TBI. When compared to the findings in TBI animals, these results indicate that HTWP acupuncture mainly enhanced neural projection and protein secretion, and the effects of HTWP acupuncture were specific because the expression of some genes, such as *Fgr*, increased without statistical significance (Figure 2D).

### HTWP Acupuncture Activates P2RX7 Dopaminergic Neurons in the vPAG

**Figure 3:**
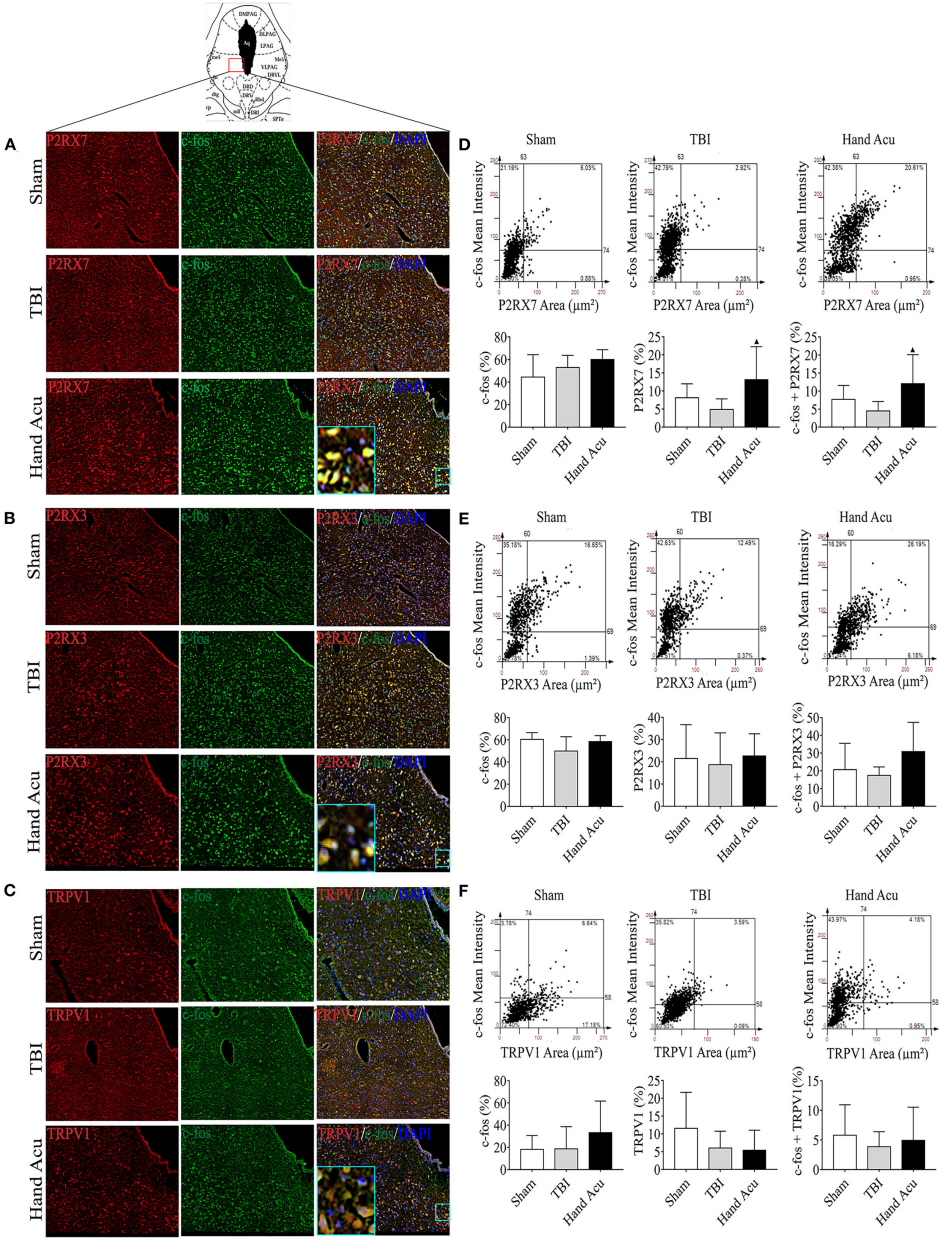
HTWP acupuncture activates P2RX7 neurons in the ventral periaqueductal gray. **(A–C)** Confocal and co-localization analyses of P2RX7, P2RX3, and TRPV1 with c-fos and DAPI. **(D–F)** HistoFAXS tissue analysis of c-fos staining mean intensity vs. area (μm^2^), and quantitative analysis by image scanning of co-localization of c-fos with P2RX7, P2RX3, and TRPV1 in the left vPAG of sham and TBI rats treated with or without HTWP acupuncture (hand Acu) (*n* = 3–4/group; one-way ANOVA). Error bars represent mean + SEM; ^▴^*P* < 0.05 vs. TBI.

We then performed histological analyses to locate the expression of P2RX7, P2RX3, and TRPV1 in the brain, particularly in the awakening-associated nucleus of the ARAS. The vPAG is located at the junction of the dorsal-ventral pathways of the ARAS (Saper et al., 2005), and vPAG neural projections express P2RX7 (Li et al., 2018), P2RX3 (Xiao et al., 2010), and TRPV1 (Li et al., 2006). Thus, we analyzed the distribution of P2RX7, P2RX3, and TRPV1 in the vPAG of sham and TBI rats without or with HTWP acupuncture treatment using confocal microscopy (Figures 3A–C) to verify that HTWP acupuncture regulates the expression of these receptors in the vPAG to reduce coma duration. We analyzed the intracellular distribution of these factors at the cellular level by co-staining for the nuclear marker DAPI and c-fos, a classical marker of neuronal activity expressed in the cytoplasm and nucleus of neurons.

P2RX7, P2RX3, and TRPV1 were mainly located in the cell membranes and cytoplasms of the vPAG neurons. Quantitative analysis of c-fos co-staining showed that the most significant effect of HTWP acupuncture in TBI rats was increased P2RX7 expression and the activation of P2RX7-expressing vPAG neurons (Figure 3D). In contrast, the activation of P2RX3-expressing neurons only slightly increased (Figure 3E), and TRPV1 neurons were not significantly activated (Figure 3F).

**Figure 4:**
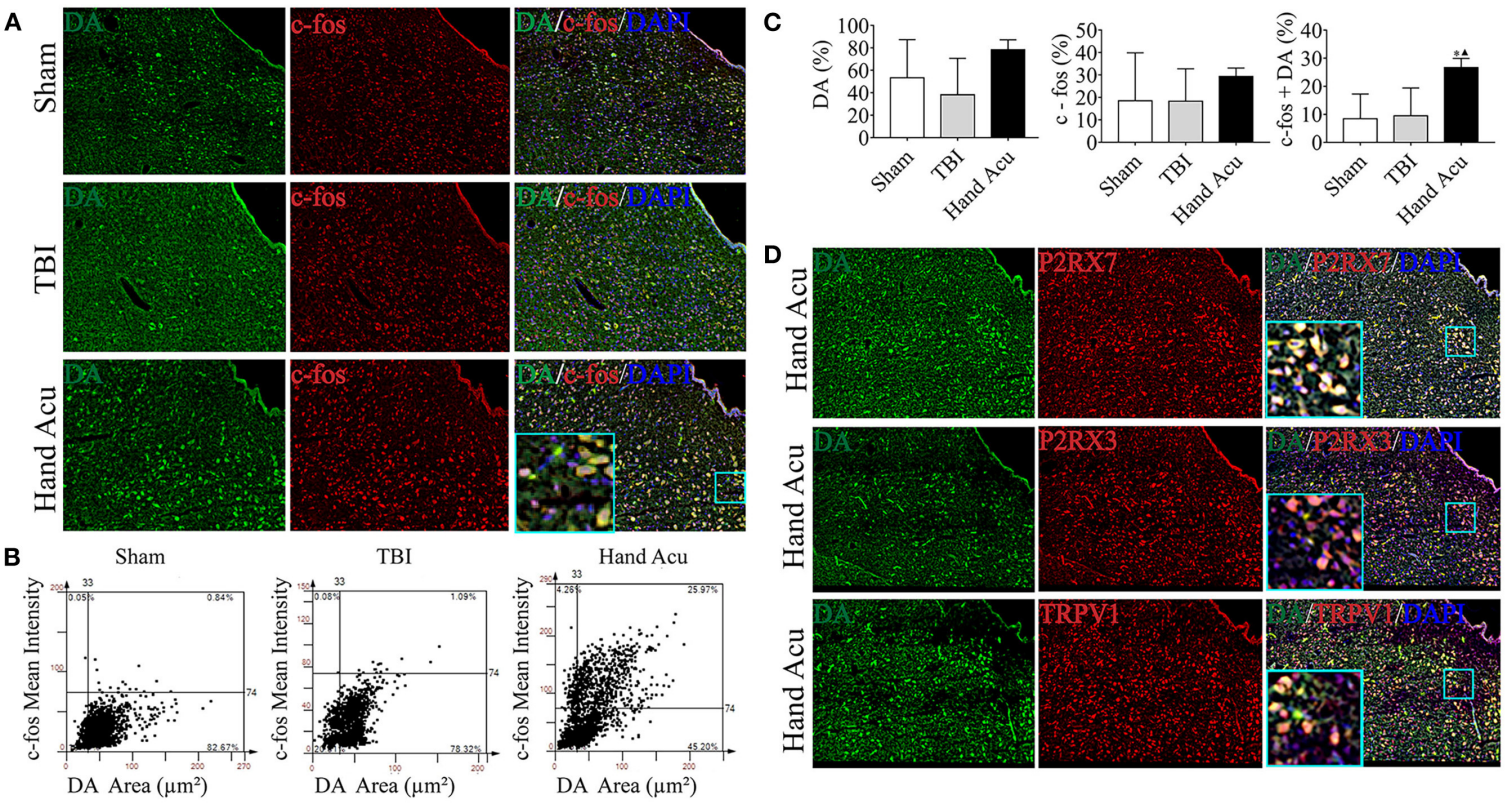
HTWP acupuncture activates dopaminergic neurons in the ventral periaqueductal gray. **(A)** Confocal and co-localization analyses of DA with c-fos and DAPI. **(B,C)** HistoFAXS tissue analysis of c-fos staining mean intensity vs. area (μm^2^), and quantitative analysis by image scanning of co-localization of c-fos with DA in the left vPAG of sham and TBI rats treated with or without HTWP acupuncture (hand Acu). **(D)** Confocal analyses and co-localization of P2RX7, P2RX3, TRPV1, and DA and in the left vPAG of sham and TBI rats treated with or without HTWP acupuncture (hand Acu, *n* = 3–4/group; one-way ANOVA). Error bars represent mean + SEM; ^▴^*P* < 0.05 vs. TBI; ^*^P < 0.05 vs. Sham.

We also revealed that there was remarkably increased *Drd3* expression (Figure 2E), which concurs with previous studies reporting the role of dopaminergic vPAG neurons in alleviating TBI (Saper et al., 2005). Thus, we hypothesized that HTWP acupuncture may alleviate TBI by activating DA neurons in the vPAG, which was verified by DA expression and neuronal activation in vPAG using confocal microscopy and colocalization with DAPI and c-fos (Figure 4A). The c-fos staining of the vPAG neurons was similar between the sham and TBI animals (18.48 ± 10.69% and 18.37 ± 7.21%, respectively), but HTWP acupuncture significantly promoted neuronal activation, as c-fos expression increased to 29.37 ± 2.15%, with an increase to 26.70 ± 1.87% in the DA area. HTWP acupuncture also induced a remarkable increase in DA staining in the vPAG of TBI animals. TBI decreased the DA expression of neurons to 38.31 ± 16.18% compared with 53.41 ± 16.98% in the sham animals, whereas HTWP acupuncture increased DA staining to 78.46 ± 5.06% (Figures 4B,C). Furthermore, our results also indicate DA excitability in the vPAG neurons expressing P2RX7, as almost all the DA neurons located in vPAG expressed P2RX7 and most also co-expressed P2RX3 and TRPV1 (Figure 4D). Therefore, HTWP acupuncture activated DA neurons and induced P2RX7 expression in TBI rats.

### HTWP Acupuncture Activates Several Neural-Projection-Related Factors in the Cortex

**Figure 5:**
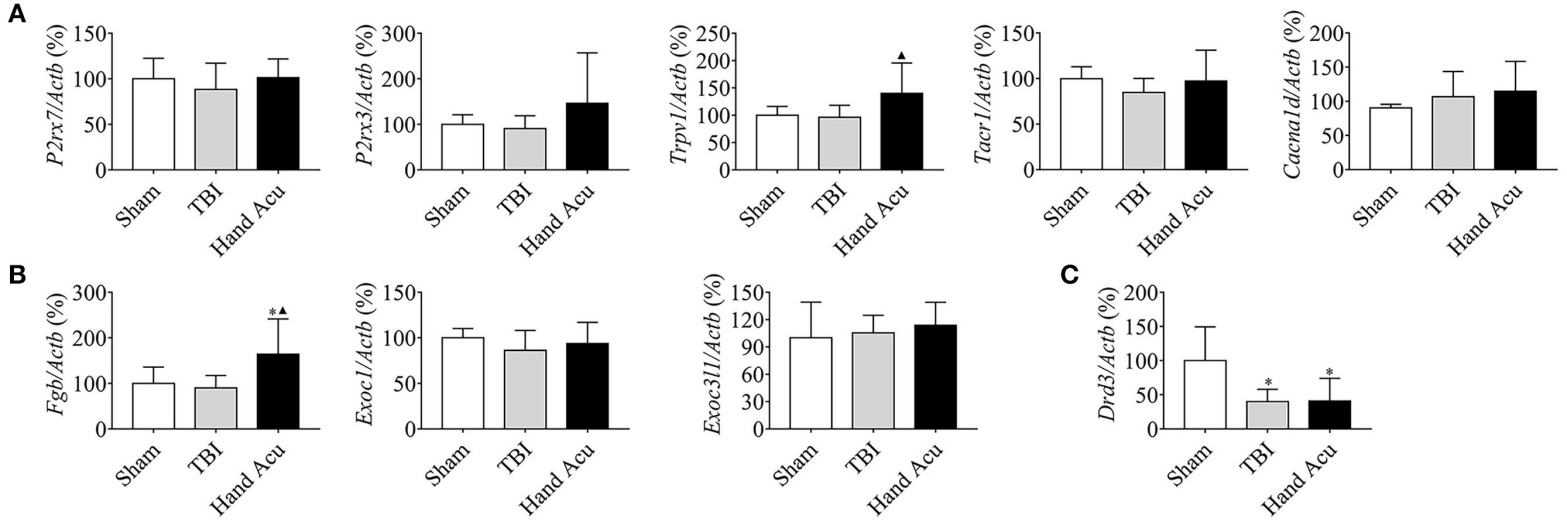
HTWP acupuncture activates several neural-projection-related factors in the cortex. Quantitative gene expression analysis of the cortex of sham and TBI rats treated with or without HTWP acupuncture (hand Acu) for genes (*n*=5/group; one-way ANOVA) related to **(A)** neural projection (*P2rx7, P2rx3, Trpv1, Tacr1*, and *Cacna1d*); **(B)** protein secretion (*Fgb, Exoc3l1*, and Exoc1); and **(C)** *Drd3*. Error bars represent mean + SEM; *P < 0.05, vs. Sham; ^▴^*P* < 0.05, vs. TBI.

The maintenance of consciousness requires the stimulation of several brain structures, such as the ventral thalamic nuclei, vPAG, and nucleus accumbens (NAc), and the cortex (Steriade and Timofeev, 2003; Luo et al., 2018). Therefore, we reasoned that the regulation of cortical neural projections may be involved in the wake-up effect of HTWP acupuncture. We performed quantitative RT-qPCR to analyze the expression of critical genes related to neural projections (*P2rx7, P2rx3, Trpv1, Tacr1*, and *Cacna1d*) and protein secretion (*Fgb, Exoc3l1*, and *Exoc1*) in the cortex of sham and TBI rats with or without HTWP acupuncture treatment. HTWP acupuncture specifically increased *Trpv1* expression by 45.20% compared with sham and TBI animals, without significantly effecting *P2rx7, P2rx3, Trac1*, and *Cacnald* expression (Figure 5A). HTWP acupuncture also increased the expression of the protein secretion gene *Fgb* by 82.14% compared with sham and TBI animals, without significantly effecting *Exoc1* and *Exoc3l1* expression (Figure 5B). In addition, we found that TBI dramatically decreased *Drd3* gene expression in the cortex by over 50%, but HTWP acupuncture did not induce a significant effect (Figure 5C). These results suggest that HTWP acupuncture can enhance neuronal projection and protein secretion in the cortex by inducing *Trpv1* and *Fgb* expression, thereby alleviating the TBI-induced comatose state.

### P2RX7, P2RX3, and TRPV1 Mediate the Wake-Promoting Effect of HTWP Acupuncture

**Figure 6:**
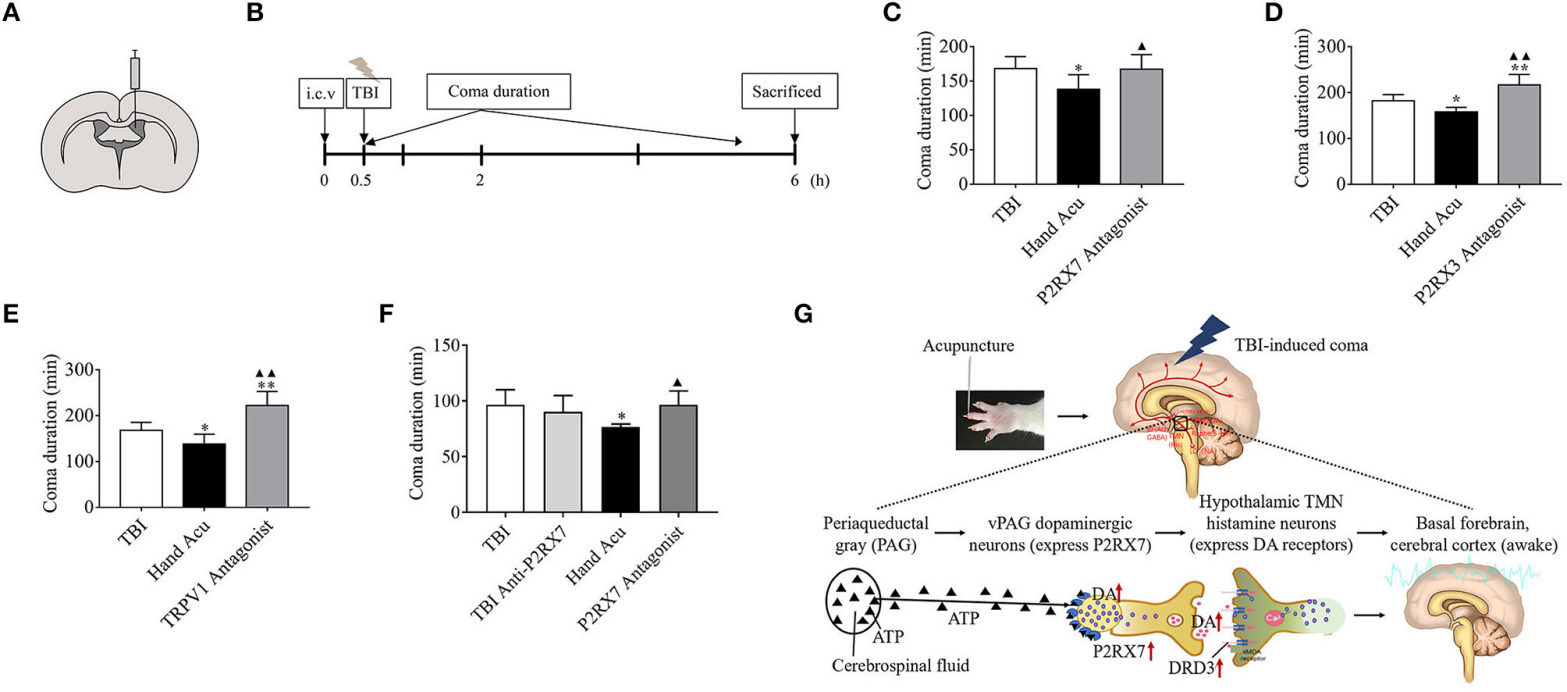
P2RX3, P2RX7, and TRPV1 mediates acupuncture-induced awakening effect in TBI rodents. **(A,B)** Experimental representation of the lateral intracerebroventricular (i.c.v) and vPAG intranuclear injection of blockers 0.5 h before starting TBI. **(C–E)** Time duration of the TBI-induced coma in control rats, treated with HTWP acupuncture (hand Acu), or pretreated with intraventricular injection of specific antagonists against **(C)** P2RX7, **(D)** P2RX3, or **(E)** TRPV1 (*n* = 6/group; one-way ANOVA). **(F)** Time duration of the TBI-induced coma in control rats, treated with HTWP acupuncture (hand Acu), or pretreated with vPAG intranuclear injection of a specific antagonist against P2RX7 (*n* = 6/group; one-way ANOVA). Error bars represent mean + SEM; *P < 0.05, **P < 0.01 vs. Sham; ^▴^*P* < 0.05, ^▴▴^*P* < 0.01 vs. TBI. **(G)** Scientific hypothesis proposing that HTWP acupuncture activates vPAG dopaminergic neurons to express P2PX7 to enhance dopaminergic connections with the hypothalamic tuberomammillary nucleus histamine neurons (expressing DRD3).

We analyzed whether the inhibition of specific factors can abrogate the potential of HTWP acupuncture to reduce coma duration. Specific factors were inhibited by performing intracerebroventricular injection of characteristic antagonists, sodium A-438079 hydrochloride hydrate, A9736 (P2RX7 antagonist), A317491 salt hydrate, A2979 (P2RX3 antagonist), and SB366791, S0441 (TRPV1 antagonist), 30 min before TBI and acupuncture (Figures 6A,B). The results show that the HTWP-acupuncture alleviation of the TBI-induced coma duration was offset by the i.c.v injection of P2RX7, P2RX3, and TRPV1 antagonists (Figures 6C–E), indicating that P2RX7, P2RX3, and TRPV1 signaling pathways mediate the awakening effect of acupuncture at HTWP. When combined with the quantitative analysis results, which showed that the most significant effect of HTWP acupuncture was P2RX7 expression induction and P2RX7 neuron activation (Figure 3D), injection of the P2RX7 antagonist into vPAG was determined to block the wakening effect of acupuncture after excluding the effect of the P2RX7 antagonist on comas (Figure 6F). Therefore, P2RX7 of vPAG could play an important role in mediating the awakening action of HTWP acupuncture.

## Discussion

On the basis of confirming that acupuncture has no promoting effect on coma caused by chloral hydrate, we found that acupuncture at HTWP and FTWP can shorten the duration of comas induced by TBI, which is consistent with the clinical records in ancient Chinese medical documents that claim that acupuncture at HTWP and FTWP can promote awakening (Li et al., 2019). There has been a plethora of modern medical studies on the ability of acupuncture at HTWP to promote consciousness from comatose states. For example, our previous experimental and clinical research confirmed that acupuncture at HTWP effectively improved comas induced by various central nervous system injuries, including TBI, stroke, and carbon monoxide poisoning (Yu et al., 2017, 2020; Zhu et al., 2019). Furthermore, our study found that acupuncture at HTWP can improve neurological deficits better than FTWP at 24 h and 48 h post-trauma. Many experimental studies have demonstrated that HTWP acupuncture effectively improves neurological deficits caused by stroke and TBI through mechanisms related to improving cerebral blood flow and tissue oxygen supply, repairing the blood–brain barrier, improving the inflammatory response, and regulating local ion balance (Miao et al., 2015; Li et al., 2019). However, there have been few experimental and clinical studies on the effect of FTWP on neurological deficit improvement, which needs further study. Therefore, in this study, we focused on the effect of acupuncture at HTWP on promoting wakefulness and improving neurological deficits.

Coma after TBI is induced via injury to the brainstem ARAS or the inhibition of its functional conduction (Parvizi and Damasio, 2003). ARAS nuclei originate from the rostral brainstem tegmentum and extend to the diencephalon, where it divides into two neural circuits, both of which project into the cerebral cortex (Saper et al., 2001). Brainstem injury produces the most profound and lasting sleepiness or even a comatose state (Gerashchenko et al., 2003). When TBI occurs, the cortex is directly impacted, which can simultaneously cause the relative displacement of brain tissue. Because there are many nerve fiber bundle connections between the cortex and brainstem, fractional nerve axons of the brainstem instantly break under the shear force, causing a coma. Additionally, a coma may also be induced by diffuse brain tissue damage after injury (Hånell et al., 2012).

Since both the ventral and dorsolateral pathways of the ARAS involve multiple critical consciousness-associated nuclei of the brainstem, we specifically screened the brainstem for potential wake-promoting pathways of acupuncture action on TBI-induced comas. Figure 1D shows that the average coma duration of the sham group was around 1.5 h, while those of the TBI group and HTWP acupuncture group were > 1.5 h; thereby, we chose 1 h as the study timepoint. According to the gene chip results, the brainstem displayed an obvious inflammatory reaction after TBI, and we speculated that axon injury may cause the activation and phagocytosis of immune cells, such as microglia, in the brain in response to the loss of necrotic tissue, thereby producing inflammation. However, excessive neuroinflammation may further damage the unbroken axons, leading to degeneration and apoptosis of the neurons and making unconsciousness more persistent and irreversible (Crowley et al., 2017). Our unpublished data showed that the inflammatory response was not significantly inhibited 1 h after acupuncture intervention, indicating that the rapid return to consciousness following acupuncture was not achieved through the inhibition of the inflammatory response. In the GO pathway analysis of the gene chip results, we found that acupuncture activated neural projection and protein secretion functions in the brainstem. Combined with previous studies that reported that acupuncture enhanced the generation and transmission of neural signals to promote wakefulness and metabolism (Ulloa et al., 2017), we speculated that the promotion of wakening by HTWP acupuncture was associated with neuronal projections and protein secretion (Yin et al., 2018).

Next, the related differentially expressed genes of neural projections and protein secretion from the GO pathway list were verified by RT-qPCR. Acupuncture was confirmed to upregulate the neural-projection-related genes *P2rx7, P2rx3, Trpv1, Tarc1*, and *Cacna1d* genes in the brainstem. Acupuncture, as a form of physical stimulation, promotes the degranulation of mast cells around acupoints and the release of ATP, thereby activating P2RX7 and/or P2RX3 at the sensory nerve endings. Subsequently, this information is transmitted to the CNS, which results in the curing of several diseases through neural-immune regulation (Díaz-Ríos et al., 2007). ATP is a neurotransmitter widely found throughout the CNS and can bind to P2X receptors to trigger cell morphological changes, the activation of intracellular signal pathways, and the release of glutamate and ATP to regulate neuron excitability (Yu et al., 2008; Li et al., 2018). TRPV1 also mediates sensory conduction, and the mechanical nociceptive stimulation produced by acupuncture activates the TRPV1 receptor in the local nerve endings of the acupoints; the signal induced by acupuncture transmits to neurons expressing TRPV1 in the CNS. The sensory information activates the calcium channels of the cell membrane, improving the excitability of the sensory neurons, and further transmits to the cortex to generate sensation (Caterina et al., 1997; Palazzo et al., 2010). Therefore, we speculated that P2RX7, P2RX3, and TRPV1 mediate the awakening effects of acupuncture by enhancing neural projection. In addition, *Tacr1* is a receptor for substance P, which is a neuropeptide related to pain and inflammation that preferentially binds to neurokinin receptor 1 (Blaine et al., 2013), while *Cacna1d* is mainly involved in neural development including neuronal migration and differentiation (Hofer et al., 2020).

We subsequently explored the key brain stem nuclei involved in the acupuncture effect mediated by P2RX7, P2RX3, and TRPV1. Several studies showed that the vPAG is an important region mediating the effects of acupuncture, i.e., treating cardiovascular (Cheng et al., 2015) and endocrine diseases (Jang et al., 2003), and analgesia (Caterina et al., 2000), whose mechanism is related to the expression of the P2X receptor in the vPAG (Liu et al., 2017). Moreover, vPAG is involved in rapid eye movement sleep gating, as the vPAG is the relay nuclei for the two ARAS pathways (Sastre et al., 1996). The present results show that P2RX7, P2RX3, and TRPV1 are expressed in the vPAG, which is consistent with previous reports showing that P2RX7/P2RX3/TRPV1 are expressed in multiple ARAS clusters, including the vPAG (Xiao et al., 2015; Madasu et al., 2016; Liu et al., 2017). Therefore, acupuncture can significantly improve the excitability of P2RX7 neurons.

DA neurons of vPAG are at the crosstalk of two arousal routes of ARAS. Severe TBI may damage the thalamocortical/corticothalamic projections from the intralaminar nuclei to junction of the frontal and posterior parietal cortices, affecting the nigrostriatal dopaminergic system (Ciurleo et al., 2013). One hour after TBI, the decreased DA level in the cerebrospinal fluid positively correlated with arousal and cognitive impairment levels (Jha et al., 2019; Kim et al., 2019), suggesting that DA neuron injury may contribute to a TBI-induced coma. Therefore, DA-mimicking drugs, such as amantadine, levodopa, and methylphenidate, can be used to treat TBI-induced comas (Tang et al., 2019). In the current study, the DA neurons were found to express P2RX7/P2RX3/TRPV1, the activities of the vPAG DA neurons were enhanced, and the expression of DRD3 in the brainstem was upregulated by acupuncture. Therefore, we speculated that acupuncture promotes the expression of the ATP receptor P2RX7 on the membrane of DA neurons, activates these neurons, then projects to the hypothalamus and basal forebrain via DA binding to increase D3 receptors in the ARAS, enhancing the release of excitatory neurotransmitters and promoting awakening. DA receptors are a family of G protein-coupled receptors (GPCRs) composed of seven transmembrane regions. At present, five types of DA receptor, D1, D2, D3, D4, and D5, have been isolated, and there have been more studies reported on the participation of D1-D3 in waking (Kebabian and Calne, 1979). D1–D3 receptors are mainly distributed in the hypothalamus, nucleus NAc, and other wake-related nuclei. NAc-D1R neurons have been shown to project into the midbrain and lateral hypothalamus, to activate the inhibitory projection neurons from NAc to the midbrain, and to promote awakening by inhibiting gamma-aminobutyric acid (GABA) neurons. This feedback mechanism of regulation can increase DA release by NAc. The activation of NAc neurons expressing D1R can induce behavioral arousal, indicating that NAc controls arousal through neuronal subsets expressing the D1 receptor (Sastre et al., 1996). Furthermore, D2 and D3 receptors are inhibitory GPCRs, and their activation inhibits neuronal activity by inducing hyperpolarization. However, D2 and D3 receptors may inhibit GABAergic interneurons, leading to the activation of projection neurons (Sheng et al., 2009). Bromocriptine, an agonist of the postsynaptic D2 receptor, improved the awakening and consciousness of five patients with TBI (Ciurleo et al., 2013). Our results preliminarily support the upregulation of D3 receptor by acupuncture, but the wake effect and mechanism of upregulating D3 and other receptors by acupuncture remains to be further verified.

The two arousal pathways of ARAS eventually project into the cerebral cortex (Saper et al., 2005). Acupuncture upregulated several neurotransmitter receptors in the damaged cortex, particularly TRPV1. Combined with the findings that TRPV1 mediates acupuncture to promote wakefulness and acupuncture upregulates the expression of TRPV1 in the cortex, we need to further verify if TRPV1-expressing neurons are activated in the cortex after receiving acupuncture information and thereby induce arousal. Moreover, the *Fgb* gene was upregulated in the brain stem and impaired cortex and was associated with increased protein secretion, but its exact role in the wake-up effect of acupuncture needs additional investigation.

After finding that P2RX7 and P2RX3 in the vPAG and TRPV1 in the cortex are regulated by acupuncture, we intraventricularly infused P2RX7, P2RX3, and TRPV1 antagonists into acupuncture-treated TBI rats to confirm the mediating roles of these signaling pathways. The results show that the P2RX7, P2RX3, and TRPV1 antagonists effectively blocked the acupuncture effect, indicating that P2RX7, P2RX3, and TRPV1 mediate the waking effect of the treatment. P2RX7, P2RX3, and TRPV1 have been reported to mediate acupuncture analgesia, whereas we found for the first time that P2RX7, P2RX3, and TRPV1 seem to promote the awakening action of HTWP acupuncture. Based on the results of the quantitative analysis, which showed that the most significant effects of HTWP acupuncture were inducing P2RX7 expression and activating P2RX7-expressing vPAG neurons in TBI rats, combined with the finding that injection of the vPAG nucleus with the P2RX7 antagonist inhibited the awakening effect of acupuncture, we speculated that P2RX7 of vPAG plays a key role in mediating wakening. This study was focused on 1 h of the acute phase, during which increased P2RX7 expression activated dopamine neurons to promote awakening. We will further examine c-fos expression in dopaminergic neurons of TBI rats subjected to vPAG intranuclear injection of the P2RX7 antagonist in a future investigation. In addition, we have observed that HTWP acupuncture has an improvement effect on neurological deficits in TBI rats at 24 and 48 h, and the exact mechanisms involved, particularly the role P2RX7 plays in improving the neuroprotection of TBI rats, are worth further study.

## Conclusion

Given the results of our previous studies that showed acupuncture at HTWP improved the consciousness level of coma patients with several CNS injuries, our present work elucidated that HTWP acupuncture may activate P2RX7-expressing dopaminergic neurons in the vPAG, increase DA production and release to enhance ARAS neural projection, and promote the awakening of TBI rats in comas. In addition, TRPV1 may play an enhanced nerve-projection role in the cortex. These findings provide innovative scientific evidence for the clinical application of acupuncture to promote awakening. Based on the above results, we put forward the following hypotheses, as shown in Figure 6G: Acupuncture at HTWP promotes wakefulness in TBI rats by exciting P2XR7-expressing DA neurons in the vPAG of the brain. Combined with the classic conduction pathway of the ARAS, acupuncture at HTWP is speculated to excite the histamine neurons of hypothalamic tuberomammillary nuclei that express dopamine receptors, which receive DA projection from the vPAG.

## Data Availability Statement

The datasets presented in this study can be found in online repositories. The names of the repository/repositories and accession number(s) can be found at: NCBI BioProject, accession no: PRJNA663342.

## Ethics Statement

The animal study was reviewed and approved by Animal Care and Use Committee of Tianjin University of Traditional Chinese Medicine.

## Author Contributions

ZX, XC, and YiG: conceptualization. HT, WL, QZ, YZ, SW, and SL: methodology and investigation. HT, SQ, and WL: statistical analysis and data curation and analysis. HT, SQ, WL, and ZX: original draft preparing and writing. YiG, YoG, BL, and YGo: review and editing. ZX and YiG: supervision, project administration, and funding acquisition. LU: contributed to the experimental design, data interpretation, figure presentation, and manuscript preparation. The manuscript was revised and approved by all the authors.

## Conflict of Interest

The authors declare that the research was conducted in the absence of any commercial or financial relationships that could be construed as a potential conflict of interest.

## Abbreviations

GO: Gene Ontology
mNSS: modified neurological severity score
NAc: Nucleus accumbens
TRPV1: transient receptor potential vanilloid 1
ARAS: ascending reticular activating system
P2RX7: ATP-gated ionotropic P2X7 receptor
P2RX3: ATP-gated P2X3 purinergic receptors
CNS: central nervous system
DRD3: dopamine receptor D3
DA: dopamine
Exoc1: exocyst complex component 1
Exoc311: exocyst complex component 3-like 1
Fgb: folate-gold-bilirubin
FTWP: foot twelve *Jing*-Well points
GPCRs: G protein-coupled receptors
GABA: gamma-aminobutyric acid
HTWP: hand twelve *Jing*-Well points
i.c.v: intracerebroventricular
RT-qPCR: reverse transcription quantitative and polymerase chain reaction
RIN: RNA integrity number
SEM: standard error of mean
TCM: traditional Chinese medicine
TBI: traumatic brain injury
vPAG: ventral periaqueductal gray.
