# Supplementary material for: P2RX7 in Dopaminergic Neurons of Ventral Periaqueductal Gray Mediates HTWP Acupuncture-Induced Consciousness in Traumatic Brain Injury
Source: Front Cell Neurosci. 2021 Jan 13;14:598198. doi: 10.3389/fncel.2020.598198 (PMC7838360; doi:10.3389/fncel.2020.598198)
Supplement: Supplementary file 1 [file Table_1.pdf]

P2RX7 in dopaminergic neurons of the ventral periaqueductal gray mediates HTWP acupuncture-induced consciousness in traumatic brain injury

Huiling Tang[1, #], Siru Qin[1, #], Wei Li[1, #], Xuyi Chen[2], Luis Ulloa[3], Qiumei Zhu[4], Baohu Liu[5], Yinan Gong[1], Yadan Zhao[1], Songtao Wang[1], Shanshan Li[1], Yongming Guo[1,6], Yi Guo[1,7,✳], Zhifang Xu[1,6,✳]

Supplemental Table 1. The gene-specific primers used in this study

Gene	Forward primer (5′→3′)	Reverse primer (5′→3′)	Product size
Actb	CCTTCCTGGGTATGGAATCCTG	CTTCTGCATCCTGTCAGCAATG	152
P2rx3	TGAAGGGTACTGCGTCAACC	CATGGAACCCTGGGAAGAGG	120
P2rx7	CTTGGAACCCTCAGTGTCCC	CCAGGTTGCAGTCCCAGTAG	126
Trpv1	AGATCCATGAACCCGAGTGC	CGATCACCTCCAGAACCGAG	132
Tacr1	ATGGACTCTGATCTCTTCCCCA	TCCATATCACCACTACGTTGCC	148
Cacna1d	TCTGTGTTCCGGTGTGTACG	AGAGCAGCAACAGCAGAGAG	127
Drd3	GTCTGAGGCTGCATCCCATT	GCTGCAGGTGTGACAAAAGG	135
Exoc1	TGCCATCAAAGAGAGCCCTG	CCAGGTGCAGGAGATGAAGG	103
Exoc3l1	ACCGAAGCCGTGAAGTACAG	CTGTCTCAGGGCTTCTCGTG	149
Fgr	AGCTTCTCTCCGATGGCAAC	ATGTGGTCGCCTCTGTTCTG	115
Fgb	AAAACGGAGGATGGACGGTC	CAATATTCGCCTGGCAAGCC	145

Supplemental Table 2. Co-expression of differentially expressed genes among sham group, TBI group and acupuncture group

GeneSymbol	sham VS TBI	TBI VS Acu
	Foldchange	Foldchange
LOC102554819	+25.10896545	-20.35582364
Dguok	+23.59157331	-25.39890233
Kcnn3	+14.06372158	-2.575019771
Rab17	+12.7407397	-8.269854687
Klk1c8 /// LOC100911764	+12.65752186	-3.310581445
Bank1	+9.290566639	-2.862524323
Grm8	+4.768353525	-2.745858439
Reg3a	+4.701432188	-3.534222152
LOC102553732	+4.701356064	-2.558798954
Spam1	+4.575485814	-3.895508788
Mx2	+3.791177326	-9.124215883
Usp49	+3.631270441	-7.766938254
Slco1a1	+3.455499897	-2.255844213
Ccl7	+3.360354833	-2.280647047
Reg3b	+3.049109204	-2.981717037
Mab21l2	+3.024504101	-2.340734216
Cenpn	+2.992187437	-2.755892521
Gpat2	+2.884098936	-5.854101852
Zbtb40	+2.851929223	-2.334138451
Ifrd2	+2.825332028	-2.206867285
Chit1	+2.704120957	-2.971875163
Comp	+2.65502853	-2.233618588
Kctd16	+2.508820147	-2.885717553
Parpbp	+2.492197407	-2.220176186

Fga	+2.458768465	-3.583778955
LOC100910650	+2.434198274	-2.091065801
Chrm2	+2.427457365	-2.667836438
Scnn1g	+2.393094239	-2.327378221
Il27ra	+2.389911572	-2.8249272
Immp2l	+2.350356742	-2.611982974
Fancc	+2.318632703	-2.648010867
Pcsk9	+2.273592176	-2.393986819
Fam26f	+2.267059995	-2.302601266
Fbln1	+2.261857497	-2.126752659
Fas	+2.227119627	-2.036580925
Pax8	+2.220091781	-2.316939585
Vnn1	+2.157482856	-12.47739629
Pomc	+2.155464616	-2.031607768
Myh6	+2.150480031	-2.132029973
Cd93	+2.111015531	-2.230232785
Klrb1a /// RGD2301395	+2.073757171	-2.412962106
Fev	+2.010602469	-5.084489341
Kcnj12	-2.003283561	+2.963446491
Crh	-2.190490813	+2.615185502
Nphp4	-2.236131393	+2.491737299
Dio2	-2.236488388	+3.286194759
Dlx5	-2.283089884	+2.889601933
Pou3f1	-2.289146205	+2.163309291
Foxg1	-2.364778907	+4.38898578
Rpe65	-2.407339867	+2.292191631
Iqck	-2.424835434	+3.226211922
Iba57 /// LOC100912081	-2.658877214	+2.274905945

LOC102547720	-2.698719582	+2.288774379
Zfp488	-2.747191732	+2.218958849
Agap3	-2.822726484	+2.320671803
Cklf	-2.865370194	+13.06871316
Slc6a13	-3.017458854	+2.213886875
LOC100910851 /// LOC100911797 /// Spink5	-3.126972593	+2.795936859
Atp2a2	-3.405450841	+5.174499752
Sim1	-3.921846947	+3.713953601
Pstpip1	-4.259145112	+4.190862719
LOC688090 /// RT1-Bb	-5.102074848	+4.814521551
Slc10a2	-8.095956628	+9.478111954
LOC102549282	-11.05961031	+4.447115294
Aqp6	-14.02835445	+5.711655521
